# Characterization of Novel *CYP2D6* Alleles across Sub-Saharan African Populations

**DOI:** 10.3390/jpm12101575

**Published:** 2022-09-24

**Authors:** Wendy Y. Wang, David Twesigomwe, Charity Nofziger, Amy J. Turner, Lena-Sophie Helmecke, Ulrich Broeckel, Ashley D. Derezinski, Scott Hazelhurst, Andrea Gaedigk

**Affiliations:** 1 Division of Clinical Pharmacology, Toxicology & Therapeutic Innovation, Children’s Mercy Research Institute, Kansas City, MO 64108, USA; 2 Sydney Brenner Institute for Molecular Bioscience, Faculty of Health Sciences, University of the Witwatersrand, Johannesburg 2193, South Africa; 3 Division of Human Genetics, National Health Laboratory Service and School of Pathology, Faculty of Health Sciences, University of the Witwatersrand, Johannesburg 2001, South Africa; 4 PharmGenetix GmbH, A-5020 Niederalm, Austria; 5 Section of Genomic Pediatrics, Department of Pediatrics, Children’s Research Institute, The Medical College of Wisconsin, Milwaukee, WI 53226, USA; 6 RPRD Diagnostics LLC, Milwaukee, WI 53226, USA; 7 Department of Biosciences, University of Salzburg, A-5020 Salzburg, Austria; 8 School of Electrical and Information Engineering, University of the Witwatersrand, Johannesburg 2001, South Africa; 9 School of Medicine, University of Missouri-Kansas City, Kansas City, MO 64108, USA

**Keywords:** personalized medicine, pharmacogenetics, pharmacogenomics, CYP2D6, African ancestry, novel alleles, haplotypes, PharmVar

## Abstract

The *CYP2D6* gene has been widely studied to characterize variants and/or star alleles, which account for a significant portion of variability in drug responses observed within and between populations. However, African populations remain under-represented in these studies. The increasing availability of high coverage genomes from African populations has provided the opportunity to fill this knowledge gap. In this study, we characterized computationally predicted novel *CYP2D6* star alleles in 30 African subjects for whom DNA samples were available from the Coriell Institute. *CYP2D6* genotyping and resequencing was performed using a variety of commercially available and laboratory-developed tests in a collaborative effort involving three laboratories. Fourteen novel *CYP2D6* alleles and multiple novel suballeles were identified. This work adds to the growing catalogue of validated African ancestry *CYP2D6* allelic variation in pharmacogenomic databases, thus laying the foundation for future functional studies and improving the accuracy of *CYP2D6* genotyping, phenotype prediction, and the refinement of clinical pharmacogenomic implementation guidelines in African and global settings.

## 1. Introduction

Cytochrome P450 2D6 (CYP2D6) is a major drug-metabolizing enzyme contributing to the bioactivation or elimination of over 20% of medications in clinical use [1,2]. Genetic variation in the *CYP2D6* gene is known to impact enzymatic activity and thus individual drug responses. The Clinical Pharmacogenetics Implementation Consortium (CPIC) has published several guidelines for CYP2D6 substrates including opioids [3], tamoxifen [4], antiemetics [5], tricyclic antidepressants [6], and SSRIs [7], underpinning the major role of *CYP2D6* genetic variation in guiding drug therapies.

The Pharmacogene Variation (PharmVar) Consortium catalogues the genetic variation of genes involved in drug metabolism using a haplotype-based system which is also known as the star (*) allele nomenclature [8,9]. This nomenclature system is widely used in the pharmacogenetic field, such as in clinical pharmacogenetic test reporting and the Association for Molecular Pathology recommendations for clinical *CYP2D6* genotyping allele selection [10], to name a few.

The *CYP2D6* gene is one of the most polymorphic pharmacogenes with over 150 star alleles catalogued to date including those identified in this study; these comprise more than 380 individual sequence variations. While some of these variants are unique to specific star alleles, many are shared among allele definitions. In addition, *CYP2D6* copies number variants (CNVs) including gene deletions, duplications, and multiplications complicate genotype analysis and test interpretation. Additionally, structural variants including *CYP2D6-2D7* and *CYP2D7-2D6* hybrid gene copies can also interfere due to the high degree of homology with the *CYP2D7* pseudogene. Hybrid gene copies can occur on their own as “singlets”, duplications, or in tandem with another *CYP2D6* gene copy. We refer to the PharmVar *CYP2D6* GeneFocus review [11] and the structural variation document on the PharmVar *CYP2D6* gene page at https://www.pharmvar.org/gene/CYP2D6 (accessed on 1 August 2022) for detailed information regarding copy number and structural variants. Of note, PharmVar provides annotations according to the Human Gene Variation Society (HGVS) nomenclature in the “variant window” as described in the “Read Me” document available on the PharmVar *CYP2D6* gene page (also see [8,11] for additional details).

As previously discussed by Nofziger and Paulmichl, *CYP2D6* genotype analysis is indeed highly complex [12]. To accurately predict the drug response of a patient, understanding which allelic variants are present in a population is crucial for testing panels to identify not only common but also more rare star alleles, especially those with variants that are likely to alter function. Additionally, a continuous effort to catalogue *CYP2D6* alleles is necessary to further improve bioinformatic tools which have been developed to call *CYP2D6* diplotypes and report them using star allele nomenclature from the NGS data [13,14,15,16,17,18,19].

Although African populations have greater genetic diversity compared to other global superpopulations, many African ethnolinguistic groups are still under-represented in pharmacogenetics and pharmacogenomic research [20,21]. As a result, there were relatively few African ancestry *CYP2D6* star alleles defined by PharmVar, including CYP2D6*17, *29, *40, and *45, to name the most prominent. We refer to African ancestry alleles as those found almost exclusively in individuals from African populations and those from populations with significant African admixture in the last 400 years. Although data are still sparse, allele frequencies may considerably vary across continental African populations and among African Americans/Afro-Caribbeans [20,22]. Since most African populations have not been systematically characterized for *CYP2D6* allelic variation, many more *CYP2D6* alleles are likely to be discovered in these under-represented populations.

Characterizing *CYP2D6* star alleles using publicly available datasets containing large numbers of African individuals [23,24] has previously been limited because of concerns regarding the sequencing quality and depth that are required to accurately interrogate the *CYP2D6* locus. The recent release of high coverage genomes from the 1000 Genomes Project cohort [25] now provides an opportunity to investigate *CYP2D6* variation in genomes from African individuals (*n* = 504) representing the Esan and Yoruba in Nigeria, Luhya in Kenya, Mende in Sierra Leone, and the Gambian Mandinka. Twesigomwe et al. [26] utilized StellarPGx that was developed by the same group [17] and other recently developed bioinformatic algorithms to investigate *CYP2D6* variation from this data resource and identify potential novel *CYP2D6* alleles. However, the majority of the predicted novel *CYP2D6* alleles lacked experimental validation. We therefore set out to fully characterize 1000 Genomes Project samples available through the Coriell Institute that were identified via StellarPGx as potentially harboring novel *CYP2D6* star alleles. Star alleles and suballeles were considered to be “novel” if they contained single nucleotide variants that were not part of any previously defined star allele in PharmVar and/or had unique combinations of variants.

The findings of this study substantially expand our understanding of *CYP2D6* allelic variation in African populations and those with African ancestry. This is a crucial step towards the development of testing and bioinformatic tools that more accurately call *CYP2D6* diplotypes and predict CYP2D6 activity in these historically underserved populations.

## 2. Materials and Methods

### 2.1. Samples

Genomic DNA from cell lines was obtained from the Coriell Institute for Medical Research (Camden, NJ, USA). The screening criteria for study samples included continental African subjects (Esan from Nigeria; Gambian from the Western Division; Luhya in Webuye, Kenya; Mende in Sierra Leone; and Yoruba in Ibadan, Nigeria) with publicly available high-depth whole genome sequencing (WGS) data collected as part of the 1000 Genomes Project dataset [25]. Thirty-six samples with 21 potentially novel *CYP2D6* alleles (i.e., possessing variants that were not present in star alleles described by PharmVar or possessing variants in novel combinations) were identified through in silico star allele calling by Twesigomwe et al. [26] using StellarPGx [17], Aldy [14], Cyrius [18], and Stargazer [17] were initially considered. Of those, 30 were selected for experimental validation using comprehensive *CYP2D6* allele characterization. Table S3 details which samples were tested by each participating laboratory.

StellarPGx v1.2.4 (https://github.com/SBIMB/StellarPGx, accessed on 17 November 2021), used in the study by Twesigomwe et al. [26], filtered out variants with quality by depth (QD) < 10 and ABHet < 0.25 (values assigned by GraphTyper, i.e., the variant caller within the StellarPGx pipeline) during star allele calling as these low-quality variants are likely to be false positives caused by read misalignments. For the 30 samples included in this study, the predicted core variants (i.e., variants defining core star alleles) had appreciable read support (i.e., QD > 10 and 0.25 ≤ ABHet ≤ 0.75). However, their respective Phred-scaled quality (QUAL) scores ranged from 90 to 255 (maximum assigned by GraphTyper). We performed experimental validation on all 30 samples as no validated minimum QUAL thresholds in the context of the *CYP2D6* locus were available at the time of this study.

### 2.2. Collaborating Laboratories

Three laboratories collaborated in experimentally validating the predicted star alleles. Each laboratory characterized selected Coriell DNA: Children’s Mercy Research Institute (Kansas City, MO, USA), PharmGenetix GmbH (Niederalm, Austria), and RPRD Diagnostics (Milwaukee, WI, USA); these laboratories are referred to from now on as CMRI, PhGx, and RPRD, respectively. Each laboratory utilized a variety of previously established methods and approaches for allele characterization as described below. For a summary of all methods used by individual laboratories, see Tables S1 and S2.

### 2.3. CYP2D6 Genotyping

CMRI and PhGx: TaqMan™-based genotyping was performed on custom-designed OpenArray™ (Thermo Fisher Scientific, Waltham, MA, USA) panels. OpenArray™ genotyping was carried out per manufacturer protocol using the OpenArray™ AccuFill™ System and run on a QuantStudio 12K Flex Real-Time PCR System (v1.2.7). Data were manually examined, and calls were obtained using the TaqMan™ Genotyper Software (v1.6.0). *CYP2D6* alleles tested by each group are summarized in Table S1.

RPRD: Genotyping was performed as previously described [27] with the Pharmaco-Scan™ Assay Kit (Thermo Fisher Scientific, Waltham, MA, USA) following manufacturer instructions. Data were analyzed using the Axiom™ Analysis Suite 3.1 (Thermo Fisher Scientific, Waltham, MA, USA) with the commercially released allele translation table (version r9; https://www.thermofisher.com/order/catalog/product/903010TS?SID=srch-srp-903010TS, accessed on 1 March 2022). *CYP2D6* alleles tested with the PharmacoScan™ are summarized in Table S1.

### 2.4. Detection of Copy Number Variation

CMRI: Samples were subjected to CNV testing by long-range PCR (XL-PCR) as previously described [27,28,29,30]. Briefly, a series of XL-PCR reactions were performed to detect the presence of CNVs. “Fragment A” (6.6 kb) amplifies the entire *CYP2D6* gene. “Fragment B” (3.5 kb) amplifies a duplication-specific product from alleles such as CYP2D6*1x2 and *2x*2*. “Fragment H” (5 kb) amplifies *CYP2D7-CYP2D6* hybrid genes (i.e., CYP2D6*13). “Fragment D” amplifies an 8.6 kb product encompassing the duplicated gene copy (CYP2D6*1x2, *2x*2,* among others) and a 10.2 kb long amplicon when the additional gene copy has a *CYP2D7-*derived downstream region (i.e., amplifies the CYP2D6*36 gene copy of a CYP2D6*36+*10). CYP2D6*5 gene deletions were also detected using XL-PCR. All reactions were performed using 2x KAPA LongRange HotStart ReadyMix with dye (Roche Diagnostics, Basel, Switzerland) and 15 ng/µL of genomic DNA (gDNA). XL-PCR primer sequences and cycling conditions are summarized in Table S2. Amplicon formation and length were determined by agarose gel electrophoresis using SYBR™ Safe DNA Gel Stain (Thermo Fisher Scientific, Waltham, MA, USA).

Samples with CNVs detected by XL-PCR were subjected to droplet digital PCR (ddPCR) to quantitatively measure copy number. ddPCR was performed as previously described [27,31] using TaqMan™-based copy number assays (Thermo Fischer, Waltham, MA, USA) with the following modification and published by Wen et al. [32]: intron 6 copy number was interrogated as a single assay and copy number of the 5′UTR, and exon 9 targets were duplexed in an amplitude-based single reaction. Droplets were generated, thermal cycled, and read using the Bio-Rad QX200 AutoDG Droplet Digital PCR System (Bio-Rad Laboratories, Hercules, CA, USA) per manufacturer protocol. Data were manually analyzed using QuantaSoft™ Analysis Pro (v1.0.596; Bio-Rad Laboratories, Hercules, CA, USA) software.

PhGx: Qualitative determination of *CYP2D6* structural variation was carried out using XL-PCR (Table S2) as previously described [30,33,34,35]. “Fragment 1” (6.6 kb) amplifies the entire *CYP2D6* gene. “Fragment 2” is indicative of a duplication and/or the presence of certain types of *CYP2D7/2D6* hybrid genes. “Fragment 3” is indicative of a full gene deletion. “Fragment 4” amplifies *CYP2D6/CYP2D7* hybrid genes, and “Fragment 5” amplifies *CYP2D7/CYP2D6* hybrid genes. All reactions were performed using JumpStart^TM^ REDAccuTaq^®^ LA DNA–Polymerase (Merck, Kenilworth, NJ, USA). Reactions were cycled as per manufacturer instructions except when PCR reactions contained 10% DMSO. PCR reactions were 15 µL and 50 µL if products were amplified for Sanger sequencing. All PCR reactions contained 3 ng/µL gDNA. Amplicon formation and length were determined using agarose gel electrophoresis with Midori Green Advanced nucleic acid stain (Biozym Scientific GmbH, Hessisch Oldendorf, Germany).

Qualitative assessment of structural variation was verified by quantification of *CYP2D6* copy number using ddPCR targeting three gene regions (5′ UTR, intron 2, and exon 9). Reactions were carried out on the QuantStudio 3D (QS3D) Digital Real-Time PCR System (Thermo Fisher Scientific, Waltham, MA, USA) using the QS3D Digital PCR 20K Chip Kit v2 and QS3D Digital PCR Master Mix v2. Briefly, 100 ng genomic DNA were digested with FastDigest *Spe*I (Thermo Fisher Scientific, Waltham, MA, USA), as recommended; 4 µL of the digestion mix were subsequently used for each ddPCR reaction, which was set up and cycled according to manufacturer instructions. Data were analyzed using the QS3D Analysis Suite^TM^ (v3.1.6-PRC-build18) at the following settings: 95% confidence level, 10% precision, and Poisson plus quantification algorithm.

RPRD: Quantitative real-time PCR reactions using four different *CYP2D6* TaqMan™ CN assays (Thermo Fisher Scientific, Waltham, MA, USA) targeting various gene regions (5′ UTR, intron 2, intron 6, and exon 9) were used as previously described [36]. Assays were performed on a CFX384 Touch Real-Time PCR Detection System (Bio-Rad, Hercules, CA, USA). Assay details for quantitative PCR for CNV determinations are provided in Table S1.

### 2.5. Allele-Specific Long-Range PCR (ASXL-PCR) and Sanger Sequencing

CMRI: To fully characterize alleles, ASXL-PCR amplicons were Sanger sequenced. Allele-specific primers (Table S2) were chosen based on the presence of heterozygous variants per WGS data from the 1000 Genomes Project (https://www.internationalgenome.org/, last accessed 12 January 2022). ASXL-PCR was performed as described above for CNV detection using XL-PCR. Briefly, XL-PCR products were purified using the QIAquick PCR Purification Kit (Qiagen, Hilden, Germany) and quantified with a NanoDrop™ One instrument (Thermo Scientific, Waltham, MA, USA). Bidirectional Sanger sequencing was carried out as previously described [37] using BigDye Terminator v3.1 chemistry and run on a 96-capillary array 3730XL DNA Analyzer (Thermo Fisher, Waltham, MA, USA). Sequence traces were analyzed using Sequencher™ software v.5.4.6. For samples with a CYP2D6*5 gene deletion (CNV = 1 copy), “Fragment A” served as the sequencing template.

PhGx: To determine all sequence variants of a haplotype, “Fragment A” amplicons were first Sanger sequenced. To phase variants detected in Fragment A, samples were subjected to ASXL-PCR and Sanger sequenced. ASXL-PCR reactions utilized by PhGx are detailed in Table S2. All PCR reactions were prepared in 50 µL reaction volumes and contained 5 ng/µL gDNA. Amplicon formation and length were determined using agarose gel electrophoresis with Midori Green Advanced nucleic acid stain (Biozym Scientific GmbH, Hessisch Oldendorf, Germany). ASXL-PCR products were purified using the QIAquick PCR Purification Kit (Qiagen, Hilden, Germany) and quantified using QIAxpert (Qiagen) as described [38]. All amplicons were commercially Sanger sequenced using Microsynth AG (Balgach, Switzerland). Sequencing primers are available upon reasonable request.

### 2.6. Single Molecule Sequencing

RPRD: To fully characterize samples with CNVs, XL-PCR amplicons encompassing the duplicated and/or nonduplicated gene copies were generated and subjected to single molecule sequencing using Nanopore as previously described [36,39]. Primers and PCR conditions used to generate the amplicons are shown in Table S2 and are as previously described [33]. Libraries were prepared using the Ligation Sequencing DNA Kit (SQK-LSK109) and Native Barcoding Kit EXP-NBD104 (Oxford Nanopore Technologies, Oxford, UK) per manufacturer recommendations. Barcoded libraries were pooled and sequenced on the MinION Sequencing instrument with a FLO-MIN106D (R9.4.1) flow cell (Oxford Nanopore Technologies, Oxford, UK) as prescribed to a minimum read depth of 10,000×). Reads were filtered for qscore >10 and base-called with MinKNOW (v21.06.13). FASTQ files were mapped to GRCh38 (NC_000022.11) using Minimap2 [40], and variant calling was performed using Nanopolish [41]. Thresholds were set as recommended (0.2). Alignments were viewed using the Integrative Genomics Viewer v.2.4.1 (IGV, Broad 153 Institute, Boston, MA, USA) aligning to the human GRCh38 reference genome to confirm genotype calls [42].

### 2.7. Allele Definitions and Designations

Alleles are described using *CYP2D6* star (*) nomenclature [11] as maintained by PharmVar [8,9] at https://www.pharmvar.org/gene/CYP2D6 (accessed on 1 August 2022) using NG_008376.4 as reference sequence (RefSeq). Variants in this report are denoted using their positions on NG_008376.4 with respect to the ATG start codon. NP_000097.3 was utilized for protein impact positions. All fully characterized novel alleles were submitted to PharmVar for designation; these alleles were released by PharmVar on 08-09-2022 (database version 5.2.3). Several star alleles previously designated were also submitted to PharmVar for independent confirmation of their definitions.

### 2.8. In Silico Function Prediction

Core variants validated in this study were evaluated using the Ensembl Variant Effect Predictor (VEP) framework [43]. The potential functional impact of these variants was predicted using seven commonly used VEP plugins, i.e., SIFT, Polyphen-2, CADD, LRT, MutationAssessor, PROVEAN, and VEST4 [44]. The variant deleteriousness thresholds used for the individual plugins were according to the ADME-specific parameters optimized by Zhou et al. (2019) [45]. We also deduced a consensus prediction of variant deleteriousness based on whether at least half of the plugins used to assess a given variant predicted it to be deleterious.

## 3. Results

### 3.1. Overview

Thirty samples were fully characterized by Sanger resequencing, genotyping, and CNV analyses. Fourteen novel star alleles were identified, of which 12 were submitted to PharmVar for designation; the other two novel alleles were independently discovered and designated as CYP2D6*146 and *149 while this investigation was underway. A summary of each Coriell sample, respective Stellar PGx predictions, and consensus diplotypes are shown in Table 1. The following sections describe the novel star alleles and suballeles found in this study as well as the alleles that were predicted to be present but not found. Lastly, we highlight alleles found among the samples, which were submitted to PharmVar as confirmatory. Table S3 comprehensively outlines all samples tested for each predicted novel allele, their consensus diplotypes (i.e., the final diplotype based on all experimental results), allele-calling algorithm outputs (StellarPGx, Aldy, Cyrius, and Stargazer outputs originally reported by Twesigomwe et al. [26]), and experimental results.

Three of the novel alleles contain nonsynonymous variants that were described in PharmVar but in novel combinations with other variants. These are indicated in Table S3. CYP2D6*146 harbors g.77G>A (p. R26H); this variant is found on other alleles including *43, where g.77G>A is the sole variant. Additionally, CYP2D6*146 has only been found as a duplication to date, which StellarPGx correctly predicted (the sample had a CNV call of 3; Table 1 and Table S3). CYP2D6*162 contains g.3854G>A (p.E410K), which is the sole core variant defining *27. Lastly, CYP2D6*154 contains g.4046G>A (p.R441H), which is the only core variant defining the *75 allele. All other novel star alleles reported in this study have variants that have not been previously described in other definitions. Table S4 details all variants found on each of the novel star alleles.

### 3.2. Novel CYP2D6 Star Alleles

**Figure 1:**
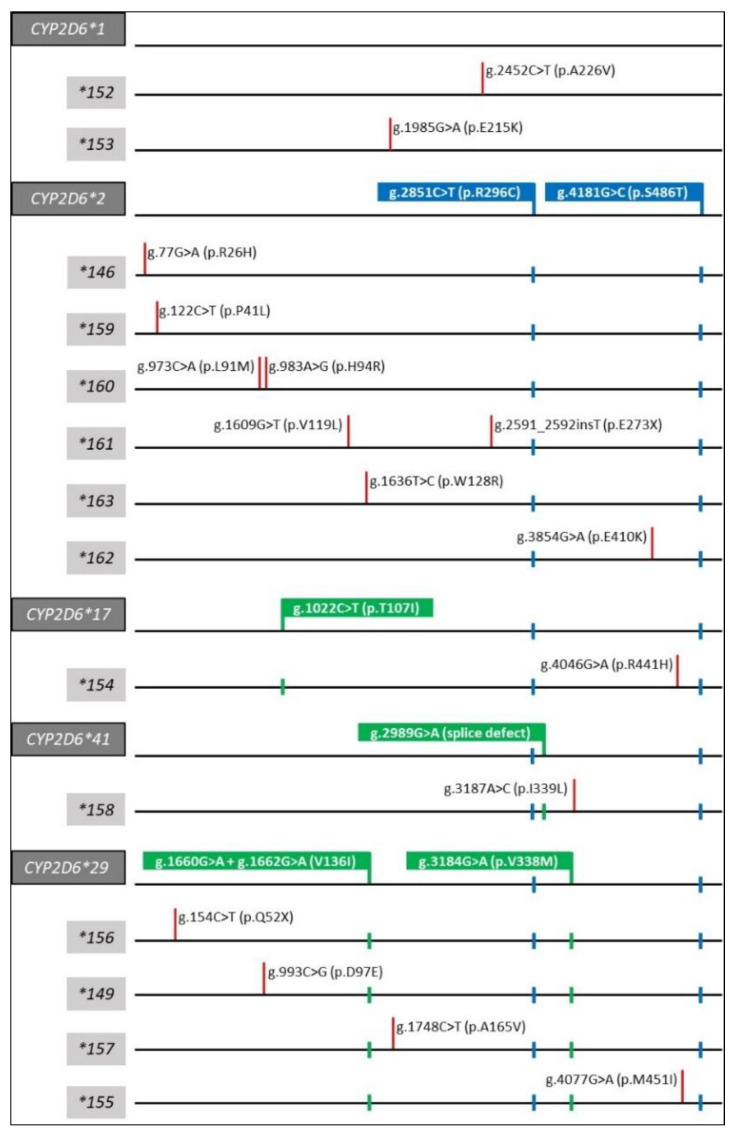
Graphical overview of the alleles characterized in this study (allele names are highlighted by grey boxes), grouped by their respective backbone alleles (highlighted by dark grey boxes). Core variants defining CYP2D6*2 are indicated by the blue boxes and subsequent markers; these represent the *2 backbone and are also found on several other alleles including CYP2D6*17, *41, and *29. Green boxes and markers represent additional core variants defining individual allelic backbones. Red markers highlight the unique variant in the StellarPGx-predicted novel allele. The unique core variants for CYP2D6*146, *154, and *162 are also present on other star alleles, such as *43, *75, and *27, respectively. Variant positions are according to the NG_008376.4 RefSeq with +1 as the first nucleotide of the translation start codon.

Fourteen novel alleles, all with GraphTyper quality scores ≥200, were experimentally confirmed. Selected StellarPGx outputs are provided in Figure S1 to illustrate the Stellar-PGx reporting of the presence of a potentially novel star allele. PharmVar designated these novel star alleles as CYP2D6*152-*163, and two matched CYP2D6*146 and *149. Figure 1 displays these alleles, grouped by their respective backbone alleles. CYP2D6*152 and CYP2D6*153 can be described as having a *1 backbone, i.e., no core variants defining other star alleles are present besides their novel variants. Six alleles, CYP2D6*146 and *159-*163 have a *2 backbone meaning that both CYP2D6*2-defining core variants (g.2851C>T, p.R296C and g.4181G>C, p.S486T) are present in these alleles in addition to other variants. Of note, one of the novel variants for CYP2D6*161 is frameshift variant g.2591_2592insT (p.E273X) and results in an immediate termination codon (TGA). CYP2D6*154 contains g.4046G>A (p.R441H) and is found on a *17 backbone, meaning that it also has the three CYP2D6*17-defining core variants g.1022C>T (p.T107N), g.2851C>T, and g.4181G>C. CYP2D6*158 has a *41 backbone meaning that it has g.2851C>T, g.2989G>A (splice defect), and g.4181G>C in addition to the novel variant at position g.3187A>C (p.I339L). Finally, CYP2D6*149 and *155-*157 are on the *29 backbone, i.e., have g.1660G>A+g.1662G>C (p.V136I), g.2851C>T, g.3184G>A (p.V338M), and g.4181G>C in addition to other variants.

#### 3.2.1. In Silico Function Prediction of Study Variants

While the star alleles with translation termination codons, CYP2D6*156 (g.154C>T; p.Q52X) and *161 (g.2591_2592insT; p.E273X), are most likely not encoding functional proteins, the functional consequences of missense variants are more difficult to predict. A set of seven in silico function prediction tools and an ADME prediction model (the consensus of CADD, LRT, MutationAssessor, PROVEAN, and VEST4) optimized for pharmacogenetic variants [44,45] were utilized to predict the functional impact of the novel variants in this study (Table 2). g.3878G>A (p.E418K) defining CYP2D6*106 was also included as this allele was found in two study samples, NA19130 and HG02970 (Table 1) and is currently described by the CPIC as having “unknown function”. Deleterious function was assigned if at least half the tools were in concordance. All seven VEP plugins and the ADME prediction model unanimously predicted the variants found on CYP2D6*154 (g.4046G>A; p.R441H), *155 (g.4077G>A; p.M451I), and *159 (g.122C>T; p.P41L) to be deleterious. All prediction tools, except LRT, indicated the g.1636T>C (p.W128R) variant found on CYP2D6*163 to also be deleterious. One of the variants defining CYP2D6*160, g.973C>A (p.L91M) met our criteria for deleteriousness with four tools predicting a loss of function; for the other variant in *160, g.983A>G (p.H94R), none of the tools predicted it to be deleterious. The g.77G>A (p.R26H) variant found on CYP2D6*146 and being the sole variant on *43 is predicted to be deleterious by only three of the seven tools, thus falling short of our criteria for deleteriousness. Additionally, g.3854G>A (p.E410K) found on CYP2D6*162 and defining *27 was predicted by all tools to be benign. Lastly, g.3878G>A (p.E418K), defining CYP2D6*106, was predicted to be deleterious by all tools except for SIFT and PROVEAN. All other variants assessed by these tools did not meet our criteria for deleteriousness.

#### 3.2.2. Novel CYP2D6 Suballeles

A total of seven novel suballeles were discovered: CYP2D6*1.048 (HG02666), *1.049 (NA19468), *2.030 (HG02561 and HG02563, parent and child, respectively), *17.005 (HG02840), *17.006 (NA19225), *45.004, and *106.003 (both found in HG02970). Per PharmVar, all alleles listed under a star number share the same core variants but differ by having additional synonymous variants or variants in noncoding regions of no or unknown functional consequence.

CYP2D6*1.048 and CYP2D6*1.049 are each defined by two variants. A G-insertion at position g.-44 was found on both suballeles; however, CYP2D6*1.048 has an additional variant in intron 4, g.2212C>T, and CYP2D6*1.049 has g.3636G>T in intron 7. Neither of these variants have been previously documented by PharmVar. CYP2D6*2.030 is a sub- allele defined by several variants including the presence of the so-called “intron 1 conversion” (a short *CYP2D7*-derived region) and g.1578C>T in intron 2, which has also not been seen on any other allele catalogued by PharmVar. CYP2D6*17.005 and *17.006 also have novel variants: g.-564G>A is in the upstream region, and g.3440G>A is in intron 7. CYP2D6*45.004 differs from previously defined *45 suballeles by having g.4027C>T in intron 8, which currently does not have a rsID. CYP2D6*106.003 has a 5bp deletion in the upstream region (g.-108_104delAAGGG), which also does not have a rsID. All variants found in these novel suballeles are detailed in Table S4.

#### 3.2.3. Predicted Alleles Not Found

Five alleles predicted to be present in 11 samples were not confirmed (Table 1). Three that were identified by StellarPGx as potentially novel had g.3280G>A (p.V370I) and g.3289G>A (p.G373S) either in *cis* (on the same chromosome) or in *trans* (on opposite chromosomes). g.3280G>A on its own defines CYP2D6*122, and g.3289G>A is part of the *6.004 suballele. It is noteworthy that g.3280G>A and g.3289G>A were not found in any of the seven samples when Sanger sequencing a *CYP2D6*-specific amplicon. Additionally, HG03485 was flagged as possibly having a novel allele due to discrepant calls from StellarPGx, Cyrius, Aldy, and Stargazer (Table S3), including a CYP2D6*36 call by Cyrius and Aldy. This sample was determined to be a CYP2D6*1.001/*36.003 and did not have any novel variants. The relatively rare CYP2D6*36 singleton was therefore correctly identified by Aldy and Cyrius. For HG03297, HG03472, and NA18867, StellarPGx called a CYP2D6*13, a rare *CYP2D7-CYP2D6* hybrid; all three samples were experimentally confirmed to be copy-neutral and not contain a hybrid allele.

## 4. Discussion

Although *CYP2D6* has been extensively investigated across populations, there is still a paucity of information regarding the extent of *CYP2D6* pharmacogenetic variation in African populations as well as in people with African ancestry. To address this knowledge gap, Twesigomwe et al. [26] investigated the distribution of *CYP2D6* star alleles and predicted phenotypes across African populations by utilizing high coverage African genome data. Although several *CYP2D6* alleles which were first discovered in participants in various Human, Heredity and Health in Africa (H3Africa) Consortium studies could be validated using single molecule long-range sequencing, many remained unconfirmed. As an extension of these efforts, this investigation aimed to fully characterize the novel alleles which were previously inferred from the 1000 Genomes Project and used available genomic DNA through the Coriell Institute.

The number of known *CYP2D6* star alleles discovered in continental African populations and those in the diaspora is relatively small considering that genetic variability is overall much higher in Africans compared to other major global populations [42]. Several *CYP2D6* alleles including CYP2D6*17 and *29 are recommended to be included in clinical testing [10] and are indeed now routinely tested owing to their frequency and contribution to the variability seen in continental Africans, African Americans, and Afro-Caribbeans. Other less frequent alleles, however, may not be included in pharmacogenetic test panels such as CYP2D6*40 and *42, both of which encode nonfunctional proteins. Still, other alleles such as CYP2D6*70, *73, etc., may not be tested at all because their function remains unknown or uncertain.

This study has led to an additional 14 African ancestry *CYP2D6* alleles (*146x2, *149, and *152.001–*163.001), several suballeles, and confirmatory allele submissions with a “definitive level of evidence” to the PharmVar database. While these novel star alleles are rare across sub-Saharan Africa (see Table S5), the combined potential impact of rare allelic variation on precision medicine strategies across Africa and in global settings may be significant. Furthermore, the allele frequencies suggesting rarity of these novel alleles should be interpreted with caution as they can be impacted by limitations in genomic data availability and sampling and may substantially differ among geographic regions and populations. We also want to point out that all 1000 genome samples come from a diverse group of speakers of Niger–Congo languages, and further studies need to not only include Niger–Congo groups but also other understudied African populations.

Many of the 14 novel alleles have the same backbones as the African ancestry decreased function alleles CYP2D6*17 and *29. One allele has the CYP2D6*41 backbone (Figure 1); CYP2D6*41 is frequently found in Africans and those with African ancestry but also in many other populations. In other words, some of the new alleles described in this study would likely have been reported according to their backbone allele if the novel alleles validated here were not tested for. Specifically, CYP2D6*154 would be reported as *17, and CYP2D6*155 would be reported as *29, etc. Many alleles are indeed assigned by “default”, a common practice which is illustrated in detail in the PharmVar CYP2D6 GeneFocus [11] and further exemplified by a case report where the phenotype of a patient was incorrectly predicted because a CYP2D6*40 allele was reported (by default) as *17 due to limited testing [46]. This case highlights the potential consequences for phenotype prediction and thus clinical care decisions if rare alleles such as those described in this report are not interrogated. These examples clearly underscore the limitations of current testing platforms, especially in understudied populations.

In other instances, results may be inconsistent among tools and test reports. For example, HG03469 (having a CYP2D6*2.019/*162.001 diplotype) was called CYP2D6*2/*27 by Aldy, “no call” by Cyrius, and flagged by StellarPGx as having a potential novel allele. These discordant results are likely due to the sample having g.3854G>A (p.E410K), which is the only variant defining CYP2D6*27. Additionally, this is an allele with an evidence level of a “limited” allele owing to its definition only being based on the exon sequence. The upstream and intron regions were not characterized. Thus, it remains to be seen if g.3854G>A exists as the sole core variant, as currently defined for CYP2D6*27 or if it is only observed in combination with other core variants. g.3854G>A is part of several alleles, namely, CYP2D6*32, *141, *148, and now *162.

In silico prediction tools may offer valuable insights into the functional impact of specific variants. The results from the tools utilized in this study suggest that some of the variants present in the novel alleles may result in considerably decreased metabolic capacity or even no activity (Table 2). For CYP2D6*154, *155, and *159, all in silico tools predicted variant deleteriousness for g.4046G>A (present on *75 and *154), g.4077G>A (unique to *155), and g.122C>T (unique to *159), respectively. Considering that the predicted deleterious variants g.4046G>A and g.4077G>A are located on CYP2D6*17 and *29 backbones, respectively, the activity of the CYP2D6*155 and CYP2D6*159 proteins is likely further diminished or eliminated altogether. In addition, the deleteriousness of g.4046G>A not only predicts the altered function for CYP2D6*155 but also *75, which is currently annotated as having an uncertain function. One limitation of the variant effect predictor (VEP) in in silico tools is that function prediction is determined on an individual variant basis and not the combination of nucleotide variants defining a star allele; in addition, these tools do not currently predict gain-of function for pharmacogenetic variants. As discussed in a recent review by Zhou et al. [47] and the references therein, the VEP tools generally underperform on pharmacogenetic variant sets. Thus, in vitro and/or in vivo functional studies are still needed to corroborate function prediction and assess the combinatorial consequences of all variants found in an allele.

Untested variants of functional significance, such as those discovered in this investigation, are likely to impact phenotype assignments and clinical care decisions and thus may contribute to adverse pharmacological outcomes for patients. As more whole genome sequence data become available for underserved populations, we anticipate that many more functionally relevant *CYP2D6* star alleles will be discovered not only in people of African ancestry but also in other understudied populations. Their subsequent validation and systematic cataloguing (in PharmVar, for example) allows for allele-calling tools such as StellarPGx, Aldy, Cyrius, and Stargazer to call *CYP2D6* diplotypes more accurately, which will result in more precise phenotype prediction.

Table S3 highlights the cases with discrepant diplotype calls by the various tools which have been resolved in this study. In addition to the PharmVar catalogue of star alleles being incomplete, another reason for false or inconsistent calls by the tools are the limitations of short-read NGS data for complex genes such as *CYP2D6*, which shares a high degree of homology with the *CYP2D7* pseudogene. Specifically, the alignment of *CYP2D7* reads onto *CYP2D6* and vice versa which may contribute to incorrect NGS variant calls affecting read depth and variant read ratios. Our data suggest that this is the case when predicted star alleles contained g.3280G>A and g.3289G>A, which are common variants found in analogous positions on *CYP2D7.* As an example, for HG02614 (CYP2D6*1.025/*17.003), read depths for these two variants were 61 reads for *CYP2D6* but only 27 reads for *CYP2D7*, suggesting that *CYP2D7*-derived reads were misaligned and mapped onto *CYP2D6*. Other examples where read misalignment may explain inconsistent allele calling include HG03297, HG03472, and NA18867 where StellarPGx falsely predicted CYP2D6*13 based on read coverage ratios while other tools did not. Our experimental validation confirmed that the CYP2D6*13 hybrid was absent in these samples. These cases highlight and underscore the need for orthogonal characterization and validation of novel allelic variants.

The contribution of read misalignments to false calls may be even more pronounced and problematic in samples with CNVs and complex structural variants containing one or more hybrid gene copies. This emphasizes the importance of access to fully characterized reference materials, including samples with copy number variation and structural variants with hybrid genes for validation purposes. Of the five confirmatory alleles submitted to PharmVar, CYP2D6*36.003 and *146.001x*2* are particularly valuable for assessing CNV detection capabilities across platforms. CYP2D6*36.003 is a *CYP2D6-CYP2D7* hybrid observed as a rare “singleton” gene copy with a *CYP2D6*-derived downstream region. The “singleton” is unique because the majority of CYP2D6*36 are found in tandem with a *10 gene copy, i.e., a CYP2D6*36+*10; in this more common arrangement, the downstream region of the CYP2D6*36 is *CYP2D7*-derived. CYP2D6*146.001 has currently only been described as a duplication (*146x*2*). The presence of two identical gene copies can impact tool diplotype calls (Table S3). The duplication could cause the incorrect phasing of not only the CYP2D6*2 core variant g.2851C>T but also g.77G>A (p.R26H), which is part of *15, *43, *46, and *148.

This study also produced *CYP2D6* genotyping information for future consideration using the Genetic Testing Reference Materials Coordination Program (GeT-RM) which develops materials for pharmacogenetic research and testing communities [48]. Lastly, the allelic variants described in this report may also be highly valuable to inform future revisions of *CYP2D6* clinical allele testing recommendations published under the leadership of the Association of Molecular Pathology (AMP) [10].

## 5. Conclusions

This study demonstrated that high coverage African whole genome sequence data can be utilized in conjunction with bioinformatic tools, such as StellarPGx, to infer novel star alleles. Although most predicted alleles were validated, some were not, which underscores the importance of validating the presence of novel nucleotide variants and star alleles discovered using bioinformatic tools with established methods. The findings presented in this study add highly valuable information regarding *CYP2D6* genetic variation in Niger–Congo language-speaking African populations and their descendants. The discovery of these alleles is one step forward in more precisely predicting CYP2D6 activity in underserved populations, which is critical for future clinical trial design, the accurate determination of the *CYP2D6* genotype to optimize the drug therapy of every patient regardless of ancestry, and to promote future precision medicine practices across world populations.

## Figures and Tables

**Table 1:** Summary of the consensus diplotype calls (reflecting all experimental testing) for each sample characterized and the corresponding novel predicted variant. StellarPGx diplotype calls shown in brackets indicate the sample may harbor a novel star allele. (The provided diplotype is the best match using current PharmVar allele definitions). See Figure S1 for an example of StellarPGx outputs. Variant positions are according to the NG_008376.4 RefSeq with +1 as the first nucleotide of the translation start codon, and amino acid positions for protein impact are according to NP_000097.3.

Sample ID	StellarPGx Call	Position	Protein Impact	rsID	Consensus Diplotype	Novel Alleles	
						Allele	Suballele
HG02807	[*17/*29]	g.4077G>A	p.M451I	rs760940331	*17.003/*155.001	*155.001	-
NA19130	[*29/*106]	g.4077G>A	p.M451I	rs760940331	*106.002/*155.001	*155.001	-
HG02666	[*1/*29]	g.993C>G	p.D97E	rs76802407	*1.048/*149.001	*149.001	*1.048
HG03442	[*2/*29] CN = 3 ^1^	g.993C>G	p.D97E	rs76802407	*2.001+*2.027/*149.001	*149.001	-
HG02840	[*17/*29]	g.154C>T	p.Q52X	rs536109057	*17.005/*156.001	*156.001	*17.005
HG02860	[*2/*29]	g.154C>T	p.Q52X	rs536109057	*2.019/*156.001	*156.001	-
HG03428	[*1/*5]	g.1985G>A	p.E215K	rs567606867	*5.001/*153.001	*153.001	-
HG03469	[*2/*2]	g.3854G>A	p.E410K	rs769157652	*2.019/*162.001	*162.001	-
HG03559	[*2/*2]	g.1636T>C	p.W128R	rs376636053	*2.019/*163.001	*163.001	-
NA18933	[*5/*29]	g.1748C>T	p.A165V	rs201006451	*5.001/*157.001	*157.001	-
NA19026	[*2/*17]	g.122C>T	p.P41L	rs373243894	*17.003/*159.001	*159.001	-
HG03313	[*17/*29]	g.4046G>A	p.R441H	rs532668079	*29.001/*154.001	*154.001	-
NA19314	[*2/*5]	g.973C>A, g.983A>G	p.L91M, p.H94R	rs28371703, rs28371704	*5.001/*160.001	*160.001	-
NA19316	[*2/*41]	3187A>C	p.I339L	rs141824015	*2.022/*158.001	*158.001	-
NA19468	[*1/*2]	g.1609G>T, g.2591_2592insT	p.V119L, p.E273X	rs374616348, rs368858603	*1.049/*161.001	*161.001	*1.049
HG02561 ^2^	*1/*2	g.77G>A	p.R26H	rs28371696	*1.024/*2.030	-	*2.030
HG02562 ^2^	*2x2/*43	g.77G>A	p.R26H	rs28371696	*1.001/*146.001x*2*	*146.001x*2*	-
HG02563 ^2^	[*2/*43] CN = 3 ^1^	g.77G>A	p.R26H	rs28371696	*2.030/*146.001x*2*	*146.001x*2*	*2.030
HG02570	[*1/*1]	g.2452C>T	p.A226V	rs140900383	*1.024/*152.001	*152.001	-
HG02614	[*1/*17]	g.3280G>A, g.3289G>A	p.V370I, p.G373S	rs61745683, rs61737946	*1.025/*17.003	-	-
HG02970	[*45/*106]	g.3280G>A, g.3289G>A	p.V370I, p.G373S	rs61745683, rs61737946	*45.004/*106.003	-	*45.004, *106.003
NA18486	[*17/*17]	g.3280G>A, g.3289G>A	p.V370I, p.G373S	rs61745683, rs61737946	*17.003/*17.003	-	-
NA19037	[*17/*29]	g.3280G>A, g.3289G>A	p.V370I, p.G373S	rs61745683, rs61737946	*17.003/*29.001	-	-
HG02645	[*17/*45]	g.3280G>A	p.V370I	rs61745683	*17.003/*45.001	-	-
NA19225	[*17/*17]	g.3280G>A	p.V370I	rs61745683	*17.002/*17.006	-	*17.006
NA19383	[*2/*2]	g.3289G>A	p.G373S	rs61737946	*2.011/*2.025	-	-
**Sample ID**	**Stellar PGx Call**		**Predicted Hybrid**		**Consensus Diplotype**	**Novel Findings**	
HG03485	[*10/*39]		*36 (*CYP2D6-CYP2D7* Hybrid)		*1.001/*36.003	-	
HG03297	(*13)+*2/*17		*13 (*CYP2D7-CYP2D6* Hybrid)		*2.019/*17.003	-	
HG03472	(*13)+*2/*29		*13 (*CYP2D7-CYP2D6* Hybrid)		*2.001/*29.001	-	
NA18867	(*13)+*2/*10		*13 (*CYP2D7-CYP2D6* Hybrid)		*2.019/*10.002	-	

^1^ CN = 3 indicates the presence of three gene copies in the sample; unknown on which chromosome the additional gene copy is located. ^2^ Samples are part of a trio (Trio ID: GB18) and were analyzed together.

**Table 2:** All variants were analyzed by in silico functional prediction tools and grouped by the star allele in which they were found. “X” indicates a deleterious call by the respective tools. Backbone alleles are shown in brackets, which are provided for reference; CYP2D6*17, *29, and *41 are designated by CPIC as decreased function alleles and *106 as uncertain function. The consensus function prediction indicates agreement of at least half the tools used to assess the variant effect. Stop-gains were, by default, considered to be deleterious.

Associated Allele [Backbone]	Variant ^1^	Consensus Function Prediction	SIFT/ SIFT Indel	Polyphen-2	CADD	LRT	Mutation- Assessor	PROVEAN	VEST4
*106 [*1]	g.3878G>A (p.E418K; rs28371733)	X	-	X	X	X	X	-	X
*146 [*2]	g.77G>A (p.R26H; rs28371696)	-	X	-	X	-	-	X	-
*149 [*29]	g.993C>G (p.D97E; rs76802407)	-	X	-	-	X	-	-	-
*152 [*1]	g.2452C>T (p.A226V; rs140900383)	-	-	-	-	-	-	-	-
*153 [*1]	g.1985G>A (p.E215K; rs567606867)	-	-	-	-	-	X	-	-
*154 [*17]	g.4046G>A (p.R441H; rs532668079)	X	X	X	X	X	X	X	X
*155 [*29]	g.4077G>A (p.M451I; rs760940331)	X	X	X	X	X	X	X	X
*156 [*29]	g.154C>T (p.Q52X; rs536109057)	X	-	-	X	-	-	-	X
*157 [*29]	g.1748C>T (p.A165V; rs201006451)	-	-	-	-	-	-	-	-
*158 [*41]	g.3187A>C (p.I339L; rs141824015)	-	-	X	X	X	-	-	-
*159 [*2]	g.122C>T (p.P41L; rs373243894)	X	X	X	X	X	X	X	X
*160 [*2]	g.973C>A (p.L91M; rs28371703)	X	X	X	-	X	X	-	-
*160 [*2]	g.983A>G (p.H94R; rs28371704)	-	-	-	-	-	-	-	-
*161 [*2]	g.1609G>T (p.V119L; rs374616348)	-	-	-	-	-	-	-	-
*161 [*2]	g.2591_2592insT (p.E273X; rs368858603)	X	X	-	-	-	-	-	-
*162 [*2]	g.3854G>A (p.E410K; rs769157652)	-	-	-	-	-	-	-	-
*163 [*2]	g.1636T>C (p.W128R; rs376636053)	X	X	X	X	-	X	X	X

^1^ Variant position is according to the NG_008376.4 RefSeq with +1 as the first nucleotide of the translation start codon; variant rs IDs are also provided; amino acid positions assessed for impact are according to NP_000097.3.

## Data Availability

Alleles reported in this study are publicly available at: https://www.pharmvar.org/gene/CYP2D6, accessed on 8 September 2022. DNA samples characterized in this study can be obtained through the Coriell Institute for Medical Research. Corresponding whole genome sequence datasets can be downloaded from the 1000 Genomes Project repositories (https://www.internationalgenome.org/data-portal/data-collection/30x-grch38, accessed on 15 September 2022).

## Supplementary Materials

The following materials can be downloaded at https://www.mdpi.com/article/10.3390/jpm12101575/s1: Figure S1: StellarPGx example output files; Table S1: methods summary for *CYP2D6* characterization by each participating laboratory; Table S2: primer list by CMRI, PhGx, and RPRD; Table S3: comprehensive result summary; Table S4: variant list of alleles; and Table S5: frequency of novel alleles.
