# Supplementary material for: Characterization of Novel CYP2D6 Alleles across Sub-Saharan African Populations
Source: J Pers Med. 2022 Sep 24;12(10):1575. doi: 10.3390/jpm12101575 (PMC9605556; doi:10.3390/jpm12101575)
Supplement: Supplementary file 1 [file jpm-12-01575-s001.zip › Supplemental Materials_FigS1_TabS1_TabS2_TabS5.pdf]

Supplementary Materials

Figure S1: Sample outputs from StellarPGx

1. Example output for HG02561 – *CYP2D6*1/*2*

```
--------------------------------------------
CYP2D6 Star Allele Calling with StellarPGx
--------------------------------------------

Initially computed CN = 2

Sample core variants:
42126611~C>G~0/1;42127941~G>A~0/1

Candidate alleles:
['1.v1_2.v1', '34.v1_39.v1’]

Result:
*1/*2

Activity score:
2.0

Metaboliser status:
Normal metaboliser (NM)
```

2. Example output for SIM001 – *CYP2D6*17/*29* (simulated sample)

```
--------------------------------------------
CYP2D6 Star Allele Calling with StellarPGx
--------------------------------------------

Initially computed CN = 2

Sample core variants:
42126611~C>G~1/1;42127608~C>T~0/1;42127941~G>A~1/1;42129132~C>T~0/1;42129770~G>A~0/1

Candidate alleles:
['17.v1_29.v1']

Result:
*17/*29

Activity score:
1.0

Metaboliser status:
Intermediate metaboliser (IM)
```

3. Example output for HG02807 – *CYP2D6*17*/[*29 + rs760940331~g.4077G>A (p.M451I)].

   This output indicates that there is a novel allele due to an additional variant, i.e., rs760940331~4077G>A (NC_000022.11:g.42126715C>T in the figure below) on either a *17* or *29* backbone. Information from other African whole genome data indicated that this variant is on the *29* backbone and this was also validated with orthogonal approaches in this study.

```
--------------------------------------------
CYP2D6 Star Allele Calling with StellarPGx
--------------------------------------------

Initially computed CN = 2


Sample core variants:
42126611~C>G~1/1;42126715~C>T~0/1;42127608~C>T~0/1;42127941~G>A~1/1;42129132~C>T~0/1;42129770
~G>A~0/1


Candidate alleles:
[17.v1_29.v1]


Result:
Possible novel allele or suballele present: interpret with caution; experimental validation
and expert review through PharmVar is recommended


Likely background alleles:
[*17/*29]

Activity score:
Indeterminate

Metaboliser status:
Indeterminate
```

4. Example output for HG03442 – *CYP2D6**2x2/[*29+ rs76802407~g.993C>G (p.D97E)].

   This output indicates that there is a potential duplication—as the copy number (CN) is three—and a novel star allele due to an additional variant, i.e. rs76802407~993C>G (NC_000022.11:g.42129799~G>C in the figure below) on either a *CYP2D6*2* or *29* backbone. Information from other African whole genome data indicated that this variant is on the *CYP2D6*29* backbone, and this was also validated with orthogonal approaches in this study.

```
--------------------------------------------

CYP2D6 Star Allele Calling with StellarPGx

--------------------------------------------


Initially computed CN = 3


Sample core variants:
42126611~C>G~1/1;42127608~C>T~0/1;42127941~G>A~1/1;42129132~C>T~0/1;42129799~G>C~0/1


Candidate alleles:
[2.v1_29.v1]


Result:
Possible novel allele or suballele present: interpret with caution; experimental
validation and expert review through PharmVar is recommended


Likely background alleles:
[*2/*29]


Activity score:
Indeterminate


Metaboliser status:
Indeterminate
```

Supplemental Table S1: Overview of the methods used by individual laboratories.

	Children's Mercy Research Institute (CMRI)	PharmGenetix (PhGx)	RPRD Diagnostics (RPRD)[1]
Genotyping	Custom OpenArray™ tests for: *CYP2D6*2, *3, *4, *6, *7, *8, *9, *19, *11, *12, *14, *15, *17, *19, *29, *31, *33, *35, *38, *40, *41, *42, *44, *45, *49, *51, *56, *59, *99, *100,* and **101.*	Custom Open Array™ test for: *CYP2D6*2, *3, *4, *6, *7, *8, *9, *10, *11, *12, *14, *17, *18, *19, *20, *29, *35, *38, *40, *41, *42, *44, *56.*	PharmacoScan™ test for: *CYP2D6*2, *3, *4, *5, *6, *7, *8, *9, *10, *11, *19, *11, *12, *14, *15, *17, *18,*19, *20, *22, *23 *25, *28, *29, *31, *33, *35, *37, *38, *40, *41, *42, *43, *44, *45, *46, *47, *48, *49, *51, *53, *54, *55, *56, *59, *62, *70, *71, *72, *73, *75, *81, *82, *84, *85, *89, *95, *100, *101, *102, *103, *109, *114*
Copy Number Determination	Qualitative XL-PCR and quantitative ddPCR (loci: 5′UTR, intron 6, exon 9)	Qualitative XL-PCR and quantitative ddPCR (loci: 5′UTR, intron 2, exon 9)	TaqMan™ (loci: 5′UTR, intron 2, intron 6, exon9)
Sequencing	ASXL-PCR + Sanger Sequencing	ASXL-PCR + Sanger Sequencing	Single molecule sequencing by Oxford Nanopore

All three laboratories used the following assays for copy number determination: 5′UTR (Hs04078252_cn), intron 2 (Hs04083572_cn), intron 6 (Hs04502391_cn), and exon 9 (Hs00010001_cn). Laboratories that employed ddPCR also used TERT (Applied Biosystems, catalogue number: 4403316) as the reference probe.

[1] Copy number determination on PharmacoScan is performed for the following 3 regions: 5′UTR, exon 9 and 3′UTR. CN is reported as Copy Number Gain (CN ≥3 detected for all 3 regions) or Loss (CN ≤1 detected for all 3 regions) or Hybrid Gain or Loss in which CN ≥3 or ≤1 is detected in only 1 or 2 of the 3 regions interrogated.

Supplemental Table S2: Summary of primer sequences used by the laboratories (CMRI, PhGx, and RPRD).

**XL–PCR Amplicons Used to Characterize Samples: CMRI**			
**Amplicon Name**	**Amplicon Description**	**Primer Sequence (5′–3′)**	**Amplicon (kb)**
Fragment A	Universal primers amplify the entire *CYP2D6* gene	F: TCACCCCCAGCGGACTTATCAACC<br>R: CGACTGAGCCCTGGGAGGTAGGTAG	6.6
Fragment B	*CYP2D6* duplication (specific for intergenic region)	F: CCATGGAAGCCCAGGACTGAGC<br>R: CGGCAGTGGTCAGCTAATGAC	3.5
Fragment H	Amplifies *CYP2D7–CYP2D6* hybrid genes	F: TCCGACCAGGCCTTTCTACCAC<br>R: CGACTGAGCCCTGGGAGGTAGGTAG	5.0
Fragment D	Amplifies entire duplicated gene: 8.6 kb with a downstream *CYP2D6*–like REP–DUP region (e.g., **1x2, *2x2*) and 10.2kb with a *CYP2D7*–like REP–DUP region (e.g., **36* of a **36+*10* tandem).	F: CCAGAAGGCTTTGCAGGCTTCAG<br>R: CGGCAGTGGTCAGCTAATGAC	8.6 or 10.2
*5 Deletion	Amplifies from the *CYP2D7*–spacer to downstream of the *CYP2D6* locus (e.g., **5* gene deletion)	F: CTCCAGCCTCCACCAGTCCAG<br>R: CAGGCATGAGCTAAGGCACCCAGAC	2.9
-2609C	Allele–specific XL–PCR using forward primer to amplify the –2609C allele	F: TGGAGAGAGGCCACCTGAGGTAGT**C**<br>R: CGACTGAGCCCTGGGAGGTAGGTAG	7.4
-2523G	Allele–specific XL–PCR using forward primer to amplify the -2523G allele	F: CGTCAAGCTTTCCGACATACAC**G**<br>R: CGACTGAGCCCTGGGAGGTAGGTAG	7.3
-2421C	Allele–specific XL–PCR using forward primer to amplify the -2421C allele	F: CCTCCCAAATCTGATGAAAAATATTAATC**C**<br>R: CGACTGAGCCCTGGGAGGTAGGTAG	7.2
-2178G	Allele–specific XL–PCR using forward primer to amplify the -2178G allele	F: GAGGCAACCTGCTCGG**G**<br>R: CGACTGAGCCCTGGGAGGTAGGTAG	6.9

-1770G	Allele–specific XL–PCR using forward primer to amplify the -1770G allele	F: CTGTCCTCAGTGGATGATCCC**G**<br>R: CGACTGAGCCCTGGGAGGTAGGTAG	6.5
-1584C	Allele–specific XL–PCR using forward primer to amplify the -1584C allele	F: CCTGGACAACTTGGAAGAACC**C**<br>R: CGACTGAGCCCTGGGAGGTAGGTAG	6.4
-1584G	Allele–specific XL–PCR using forward primer to amplify the -1584G allele	F: CCTGGACAACTTGGAAGAACC**G**<br>R: CGACTGAGCCCTGGGAGGTAGGTAG	6.4
-740C	Allele–specific XL–PCR using forward primer to amplify the -740C allele	F: TGTGTGTGAGAGAGAATGTGTGC**C**<br>R: CGACTGAGCCCTGGGAGGTAGGTAG	5.5
842G_F	Allele–specific XL–PCR using forward primer to amplify the 842G allele	F: TAGGACCTGTAGTCTGGGG**G**<br>R: CGACTGAGCCCTGGGAGGTAGGTAG	4.0
842G_R	Allele–specific XL–PCR using reverse primer to amplify the 842G allele	F: TCACCCCCAGCGGACTTATCAACC<br>R: GCCTCTTGTCAAGCCAGGATC**C**	2.8
4713G	Allele–specific XL–PCR using reverse primer to amplify the 4713G allele	F: TCACCCCCAGCGGACTTATCAACC<br>R: AGGTAGCCCTGGCCTATAGCTCC**C**	6.6
4723G	Allele–specific XL–PCR using reverse primer to amplify the 4723G allele	F: TCACCCCCAGCGGACTTATCAACC<br>R: CTGGGAGGTAGGTAGCCCTGAC**C**	6.6
**XL–PCR Amplicons Used to Characterize Samples: PhGx**			
**Amplicon Name**	**Amplicon Description**	**Primer Sequence (5′–3′)**	**Amplicon (kb)**
Fragment 1	Universal primers amplify the entire *CYP2D6* gene (same as those used by the CMRI group to generate Fragment A)	F: TCACCCCCAGCGGACTTATCAACC<br>R: CGACTGAGCCCTGGGAGGTAGGTAG	6.6

Fragment 2	Amplifies if *CYP2D6* gene duplication is present	F: GCCACCATGGTGTCTTTGCTTTCCTGG<br>R: CCGGATTCCAGCTGGGAAATGCG	9.5
Fragment 3	Amplifies if *CYP2D6* gene deletion is present	F: CACACCGGGCACCTGTACTCCTCA<br>R: CAGGCATGAGCTAAGGCACCCAGAC	3.2
Fragment 4[1]	Amplifies if *CYP2D6*/*CYP2D7* hybrid gene is present	F: TCACCCCCAGCGGACTTATCAACC<br>R: TACGGTGGGCTCCCTGCGAG	6.6
Fragment 5	Amplifies if *CYP2D7*/*CYP2D6* hybrid gene is present	F: CCTGTGTGGGCTTGGGGAGCTTG<br>R: TGTGGTGAGGTGACGAGGCTGA	5.7
-1770G	Allele–specific XL–PCR using forward primer to amplify the -1770G allele	F: CTGTCCTCAGTGGATGATCCC**G**<br>R: CGACTGAGCCCTGGGAGGTAGGTAG	6.5
-1584C	Allele–specific XL–PCR using forward primer to amplify the -1584C allele	F: CCTGGACAACTTGGAAGAACC**C**<br>R: CGACTGAGCCCTGGGAGGTAGGTAG	6.3
842G_F	Allele–specific XL–PCR using forward primer to amplify the 842G allele	F: TAGGACCTGTAGTCTGGGG**G**<br>R: CGACTGAGCCCTGGGAGGTAGGTAG	3.9
842G_R	Allele–specific XL–PCR using reverse primer to amplify the 842G allele	F: TCACCCCCAGCGGACTTATCAACC<br>R: GCCTCTTGTCAAGCCAGGATCC	2.7
Fragment 6	Amplifies entire duplicated gene: 10.1 kb with a downstream *CYP2D6*–like REP–DUP region (e.g., **1x2*, **2x2*) and 11.7 kb with a *CYP2D7*–like REP–DUP region (e.g., **36* of a **36*+**10* tandem)	F: TCACCCCCAGCGGACTTATCAACC<br>R: CGGCAGTGGTCAGCTAATGAC	10.1 or 11.7
Fragment 7[2]	Amplifies 3′ *CYP2D6* gene duplication in arrangements and tandem arrangements with some *CYP2D7*/*CYP2D6* hybrids	F: CCCTGGGAAGGCCCCATGGAAG<br>R: TAGGTAGCCCTGGC[C]TATAGCTCCCTGACGCC<br>R: TAGGTAGCCCTGGC[A]TATAGCTCCCTGACGCC	12.1
	**XL–PCR Amplicons Used to Characterize Samples: RPRD**		
**Amplicon Name**	**Amplicon Description**	**Primer Sequence (5′–3′)**	**Amplicon (kb)**
Fragment A	Amplifies non–duplicated *CYP2D6* gene copy and alleles with gene deletions	F: GTCCCACACCAGGCACCTGTACT<br>R: GAATTAGTGGTGGTGGGTGTTTG	15.6

Fragment B	Amplifies the 5′ *CYP2D6* gene copy in duplication arrangements	F: TCACCCCCAGCGGACTTATCA<br>R: CCACAGCCCTCAATAAGTGAA	12.0
Fragment C	Amplifies the 3′ *CYP2D6* gene copy in duplication arrangements	F: CCCTGGGAAGGCCCCATGGAAG<br>R: TAGGTAGCCCTGGCCTATAGCTCCCTGACGCC	12.1
Fragment D	Amplifies *CYP2D6* gene copies with *CYP2D6*–like up and downstream regions	F: TTGCCACATTATCGCCCGTGAAA<br>R: TAGGTAGCCCTGGCCTATAGCTCCCTGACGCC	8.4
Fragment E	Amplifies *CYP2D6*/*CYP2D7* hybrid genes	F: TCACCCCCAGCGGACTTATCA<br>R: TACGGTGGGCTCCCTGCGAG	6.7
Fragment G	Amplifies *CYP2D7*/*CYP2D6* hybrid genes	F: CCTGTGTGGGCTTGGGGAGCTTG<br>R: TGTGGTGAGGTGACGAGGCTGA	5.7
Fragment J	Amplifies *CYP2D7*/*CYP2D6* hybrid genes	F: CACACCGGGCACCTGTACTCCTCA<br>R: CAGGCATGAGCTAAGGCACCCAGAC	9.5

Bolded bases denote the SNP utilized for allele–specific amplification, i.e., the allele with the shown nucleotide was amplified. The underlined bases denote an intentional sequence mismatch to enhance primer specificity. ‘F’ and ‘R’ denote forward and reverse primers. Variant positions are in respect to the ATG translation start codon with the ‘A’ being +1 on the NG_008376.4 reference sequence.

[1] Modified from Kramer et al. (2009) [34], ‘ACC’ bases were added to the 3′ end of the forward primer.

[2] XL–PCR contains two reverse primers to capture both alleles of a common variant, g.4723C/A.

Table S5. Frequency of novel star alleles characterised in this study across sub-Saharan Africa (SSA)

**_CYP2D6_ Star Allele**	**Allele Details**	**Allele Count (in unrelated participants)**	**Allele Frequency in SSA (%) (N=961 participants)**
_*146x2_	Duplication of<br>*2 + g.77G>A (p.R26H; rs28371696)	1	0.052
_*149_	_*29_ + g.993C>G (p.D97E; rs76802407)	5	0.26
_*152_	_*1_ + g.2452C>T (p.A226V; rs140900383)	1	0.052
_*153_	_*1_ + g.1985G>A (p.E215K; rs567606867)	1	0.052
_*154_	_*17 + g.4046G>A (p.R441H; rs532668079)_	1	0.052
_*155_	_*29_ + g.4077G>A (p.M451I; rs760940331)	2	0.104
_*156_	_*29_ + g.154C>T (p.Q52X; rs536109057)	2	0.104
_*157_	_*29_ + g.1748C>T (p.A165V; rs201006451)	1	0.052
_*158_	_*41_ + g.3187A>C (p.I339L; rs141824015)	4	0.208
_*159_	*2 + g.122C>T (p.P41L; rs373243894)	1	0.052
_*160_	*2 + g.973C>A (p.L91M; rs28371703) +<br>g.983A>G (p.H94R; rs28371704)	1	0.052
_*161_	*2 + g.1609G>T (p.V119L; rs374616348) +<br>g.2591_2592insT (p.E273X; rs368858603)	4	0.208
_*162_	*2 + g.3854G>A (p.E410K; rs769157652)	1	0.052
_*163_	*2 + g.1636T>C (p.W128R; rs376636053)	1	0.052

The allele counts are based on findings by Twesigomwe et al. (2022) [26]. Variant positions are according to the NG_008376.4 RefSeq with +1 as the first nucleotide of the translation start codon and amino acid positions for protein impact are according to NP_000097.3.
